# Effects of *Limosilactobacillus reuteri* ID-D01 Probiotic Supplementation on Exercise Performance and Gut Microbiota in Sprague-Dawley Rats

**DOI:** 10.1007/s12602-024-10257-9

**Published:** 2024-04-18

**Authors:** Ye-Ji Jang, Han Sol Choi, Ikhoon Oh, Jae Hyuk Chung, Jin Seok Moon

**Affiliations:** 1 YUNOVIA Co., Ltd, Hwaseong, 18449 Republic of Korea; 2 Ildong Pharmaceutical Co., Ltd, Seoul, 06752 Republic of Korea

**Keywords:** Endurance exercise, *Limosilactobacillus reuteri*, Muscle fatigue, Oral probiotics, Short-chain fatty acids, Sprague–Dawley rats

## Abstract

The gut microbiota composition in animals and humans has recently been found to be influenced by exercise. Although *Limosilactobacillus reuteri* strains have notable probiotic properties that promote human health, understanding of its effects in combination with exercise and physical activity is limited. Therefore, this study examined the effects of *L. reuteri* ID-D01, a human-derived probiotic, on exercise performance and fatigue in Sprague-Dawley rats. Organ weight, maximal running distance, serum biochemistry, muscle performance, microbial community composition, and short-chain fatty acid (SCFA) levels were assessed. Results indicated that ID-D01 supplementation significantly improved endurance performance. Rats in the probiotic group demonstrated a significant increase in maximal running distance compared with that in the control group (*p* < 0.05). Additionally, levels of fatigue markers, such as lactate and creatine phosphokinase, were significantly reduced in the ID-D01-administered groups, suggesting its potential to alleviate exercise-induced fatigue. Microbiome analysis revealed a distinct shift in gut microbiota composition in response to ID-D01 administration. The group that received ID-D01 probiotics exhibited a significant increase in the abundance of SCFA-producing bacteria, particularly *Akkermansia* spp., compared with that in the control groups. Furthermore, they showed elevated production of SCFAs, such as acetate and butyrate. In conclusion, this study demonstrated that ID-D01 can enhance exercise performance and reduce fatigue. Herein, we highlighted that human-derived probiotics could improve physical performance, as observed by changes in gut microbiota composition and SCFA production.

**Supplementary Information:**

The online version contains supplementary material available at 10.1007/s12602-024-10257-9.

## Introduction

The human gut microbiota comprises trillions of microbial cells belonging to different taxa, including archaea, eukaryotes, and viruses. This complex community exhibits extensive metabolic capacity, exceeding three million genes, and performs a critical function in maintaining general health and averting diseases [1, 2]. Intrinsic and extrinsic factors, such as age, probiotic and antibiotic use, and dietary habits, contribute to the dynamic and individualized nature of the gut ecosystem [3]. Recent research has highlighted exercise as a significant behavioral factor that influences gut microbiota composition in both animals and humans. Minimal levels of physical activity can even cause changes in the composition of the gut microbiota [4]. Athletes who exhibit higher alpha diversity of gut bacteria may attribute this to increased protein intake, which benefits tissue repair, dietary energy use, carbohydrate metabolism, cellular structure, and nucleotide biosynthesis [5, 6]. The reciprocal relationship between exercise and gut microbiota is notable, with exercise-inducing changes in gut microbiota composition, and gut microbiota composition conversely influencing exercise performance. Microbiota composition and metabolic activities within the digestive tract participate in the fermentation of indigestible polysaccharides, producing short-chain fatty acids (SCFAs), such as acetate, propionate, and butyrate [7]. Acetate and propionate are substrates for mitochondrial oxidation, lipid production, and gluconeogenesis. Propionate acts as a precursor for hepatic glucose synthesis [8]. Butyrate, transported from colonic epithelial cells to mitochondria, functions as an energy source after being converted into acetyl-CoA through fatty acid oxidation under aerobic conditions. Subsequently, the compound enters the tricarboxylic acid cycle and generates NADH, ATP, and CO_2_ via the electron transport chain [9]. SCFAs produced by gut microbiota have a substantial influence on energy metabolism during physical activity, facilitating exercise-induced adaptation and functioning as energy providers for the liver and muscle cells, thereby enhancing endurance performance [10].

Nutritional supplements are frequently used to promote athletic performance, facilitate training adaptation, and enhance post-exercise recuperation [11]. Probiotics, which are recognized as a simple way to positively affect the gut microbiota, are increasingly used by athletes. Probiotics are live, nonpathogenic microorganisms that balance the microbiome and exert beneficial effects on the host. These include various bacterial species, with the most significant genera being *Lactiplantibacillus* and *Bifidobacterium*. Studies have indicated that probiotic supplementation can enhance exercise performance and facilitate fatigue recovery [12, 13]. However, differences in effectiveness may arise among various strains, and bacteria from different sources may vary in potency and their ability to colonize within the host [14]. Historically, probiotics were derived from fermented foods. However, contemporary interest now centers on human probiotic strains that are found in the human digestive tract and are free from human byproducts [15]. Recent in vitro and animal studies have shown that human-derived probiotics exhibit superior results compared with those of their plant- and dairy-based counterparts [16]. Consequently, interest in developing and employing human-based probiotics is growing [17].

*Limosilactobacillus reuteri* is a microorganism commonly used to promote human health because of its valuable probiotic characteristics. This extensively researched probiotic provides health benefits through various metabolic mechanisms, such as enhancing the production of anti-inflammatory cytokines [18, 19] and regulating intestinal microbiota by promoting the production of antimicrobial molecules, such as reuterin [20]. Its effects on body weight and obesity [21], insulin sensitivity and glucose homeostasis [22], gut integrity [23], immune regulation [24], and liver dysfunction [25] have been demonstrated. However, studies on its effects of exercise and physical activity are limited. Therefore, this study aimed to evaluate the benefits of humanized *L. reuteri* ID-D01 derived from human feces in improving exercise performance and exerting anti-fatigue effects in Sprague–Dawley rats. In addition, changes in the gut microbiota were investigated to elucidate the potential mechanisms involved.

## Materials and Methods

### Preparation of *Limosilactobacillus reuteri* ID-D01

The *L. reuteri* ID-D01 (ID-D01) strain was isolated from human feces with the approval of the Institutional Review Board of Seoul National University, Bundang Hospital (Seongnam, South Korea) (IRB accession number: B-1605-345-003). For in vivo sample preparation, ID-D01 was cultured in de Man, Rogosa, and Sharpe broth (BD Difco, MD, USA) at 37 °C. The culture medium was removed via centrifugation at 9,680 x *g* for 15 min at 4 °C, and the cell pellets were collected and pre-frozen at − 80 °C for 24 h. The pre-frozen pellets were then lyophilized using a freeze dryer (Ilshin BioBase, Seoul, South Korea) and mixed with dried starch (Samyang, Seoul, South Korea) to produce lyophilized pellets for oral administration. Low- and high-dose concentrations of the test substances were 3.6 × 10^7^ CFU/g (Dose Low; DL) and 1.82 × 10^9^ CFU/g (Dose High; DH), respectively.

### Experimental Design for the Animal Study

Five-week-old Sprague-Dawley rats (*n* = 32) were obtained from Samtako Inc. (Osan, South Korea) and housed under controlled conditions (21 ± 2 °C, 50 ± 20%, 12-h light/dark cycle). Animal experiments were performed in accordance with approval from the Institutional Animal Care and Use Committee (IACUC) of Chungbuk National University (CBNUA-1718-22-01). After a seven-day adaptation period, the animals underwent a five-day treadmill acclimatization exercise, except for those in group G1. Treadmill acclimatization consisted of the following speeds: On day one, the rats exercised at a speed of 10 m/min for 10 min, followed by 15 m/min for 10 min on day two, 20 m/min for 10 min on day four, and a final session of 20 m/min for 15 min. Following this, the rats were separated into four groups based on body weight using a Z-arrangement: non-training group (G1), training group (G2), training + DL (G3), and training + DH (G4), with eight rats per group.

**Fig. 1:**
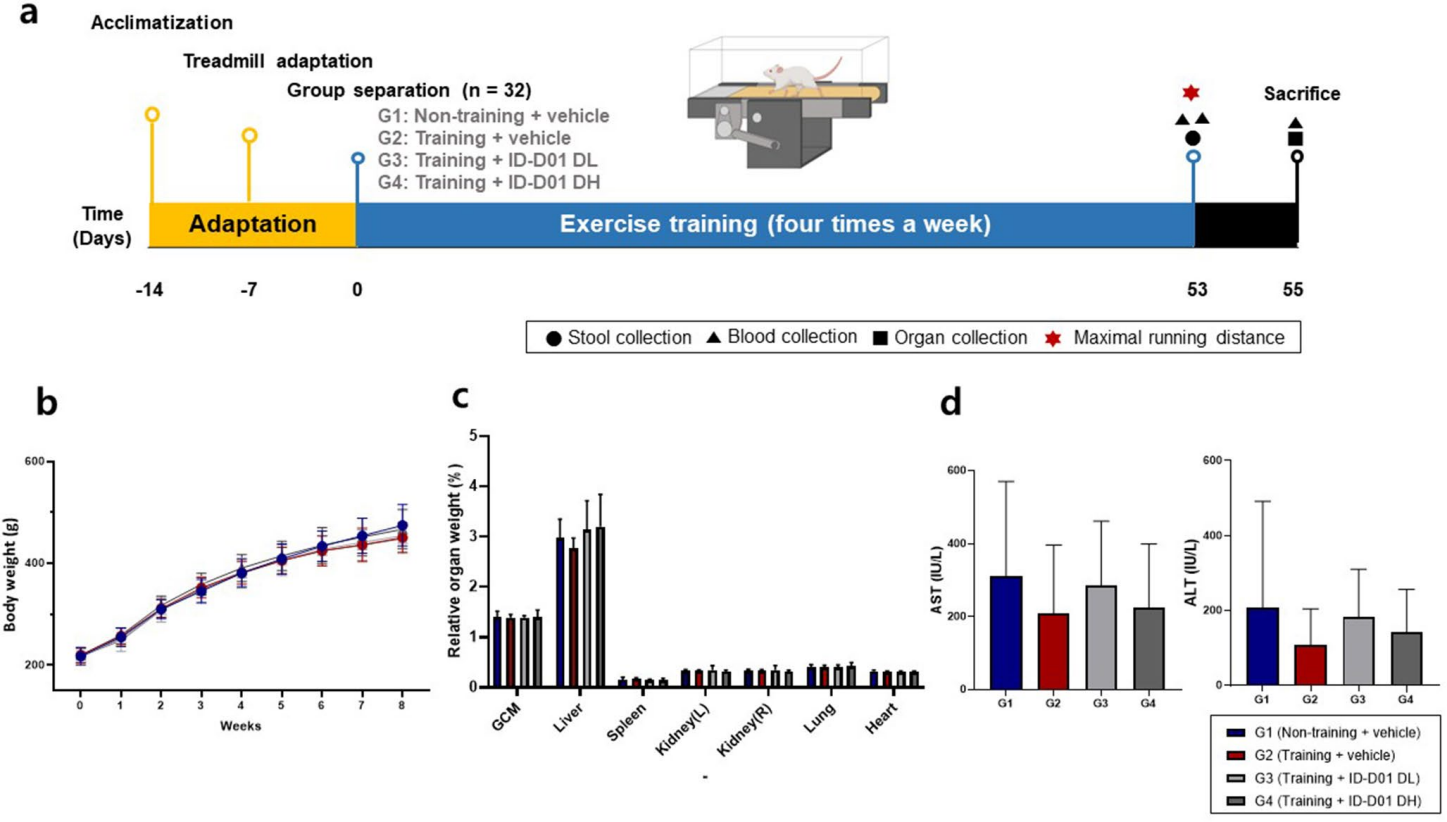
Impact of oral probiotic *Limosilactobacillus reuteri* ID-D01 on general characteristics of the Sprague–Dawley rat. Experimental design (**a**), body weight changes (**b**), organ weights (**c**), and serum alanine transaminase (ALT) and aspartate transaminase (AST) levels (**d**). DH = high dose; DL = low dose; GCM = gastrocnemius muscle

The probiotics were orally administered for 53 days using starch (0.2 g/mL) in phosphate buffer solution (PBS; pH 7.0) as a vehicle for G1 and G2. The G3 and G4 treatment groups received oral doses of 10 mL/kg probiotics in PBS (pH 7.0). The G2-G4 training groups engaged in graded exercise training four times per week throughout the administration period. Initially, they exercised at a 0° incline, 10 m/min speed, and 20 min duration on day one. This gradually progressed to a 14° incline, 24 m/min speed, and 45 min duration in the final 51 days. The maximum running distance was recorded on day 53, and the rats sacrificed on day 55. Body weight, food consumption, and water intake were recorded weekly. Organs, blood, and feces were collected for further analyses (Fig. 1a).

### Organ Weights

On day 55, the liver, spleen, kidneys, heart, lungs, and gastrocnemius muscles (GCMs) were harvested after an 18-h fasting period and blood sampling. The relative weight of each organ was calculated using the following formula: Relative organ weight (%) = organ weight (g)/pre-autopsy body weight (g) × 100 [26]. The GCM samples were immediately frozen in liquid nitrogen and stored at − 80 °C for subsequent analysis.

### Estimating the Maximal Running Distance

The maximal running distance was measured for all groups under uniform treadmill conditions, including a 14° incline, speed of 25 m/min, and an electric shock intensity of 0.6 mA. The total exercise time to exhaustion was recorded for each individual, and exhaustion defined as the point at which a rat remained on the electric grid without responding to the electric shock for more than 10 s.

### Analysis of Serum Biochemistry

Blood samples were obtained from all groups (G1-G4) thrice before, after, and two days after maximum running activity. Serum was segregated from the collected blood and preserved at − 80 °C. Serum glucose (GLU), aspartate transaminase (AST), alanine transaminase (ALT), blood urea nitrogen (BUN), creatinine (CREA), lactate dehydrogenase (LDH), and creatine phosphokinase (CPK) levels were measured using an AU480 analyzer (Beckman Coulter, Krefeld, UK). Lactate (LAC) and ammonia concentrations were measured using a 7180 analyzer (Hitachi, Tokyo, Japan).

### Enzyme-linked Immunosorbent Assay

The LAC, citrate synthase (CS), and glycogen contents in GCM tissues stored at − 80 °C were assessed using enzyme-linked immunosorbent assay kits, according to the manufacturer’s instructions. A colorimetric l-Lactate Assay Kit (Abcam, Cambridge, UK) was used to examine LAC levels. CS activity and glycogen contents were quantified using a Citrate Synthase Assay Kit (Abcam) and colorimetric Glycogen Assay Kit II (Abcam), respectively.

### Microbial Community Analysis

The microbiome composition of Sprague-Dawley rats was examined through 16S rRNA gene sequencing. The manufacturer’s instructions were followed when performing genomic DNA extraction, which was done using the PowerSoil DNA Isolation Kit (Cat. no. 12,888; MO BIO, Carlsbad, CA, USA). Illumina Sequencing Library protocols were used to process each sample before sequencing the amplified DNA with the V3–V4 region on a MiSeq platform (Illumina, San Diego, CA, USA) at Macrogen (Seoul, South Korea). Microbiome analysis was performed using the 16 S rRNA gene-based Microbial Taxonomic Profiling platform from the EzBioCloud App (CJ Bioscience, Seoul, South Korea). The 16S rRNA database (DB ver. PKSSU4.0) enabled taxonomic assignment of the 16S rRNA amplicon reads. Both alpha and beta diversity were assessed, and the ACE, Chao1, Shannon, and phylogenetic diversity indices used to measure alpha diversity. Beta diversity was examined using Jensen-Shannon distance matrices. Principal coordinate analysis (PCoA) was used to analyze variations between the sample groups. The linear discriminant analysis (LDA) effect size (LEfSe) method was used to differentiate the bacterial biomarkers represented in different groups at varying taxonomic levels.

### Short Chain Fatty Acids Analysis

To extract SCFAs, each fecal sample (1.0 g) was thawed, suspended in 8 mL water, and sonicated for 30 min using a UCP10 sonicator (Lab Companion, Daejeon, South Korea). The samples were then kept at room temperature for 24 h. The solution was then centrifuged at 9,000 rpm for 5 min at 4 °C, and the resulting supernatant filtered through a 0.45-µm filter. Subsequently, a 10 µL filtrate sample was injected into a Waters e2695 HPLC system, which had a Waters 2998 UV detector. Chromatography was performed using a mobile phase containing 5 mM sulfuric acid in water under isocratic elution conditions. A Coregel 87H3 column (7.8 × 300 mm, 5 μm) (Concise Separations, San Jose, CA, USA) was used for this purpose, and the detection wavelength set to 210 nm. Other chromatography parameters involved maintaining the temperature of a column oven at 35 °C, flow rate at 0.6 mL/min, and a run time of 60 min. Individual SCFAs (acetic, propionic, and butyric acids) were quantified and identified using known standard retention times and peak areas after chromatographic separation. Standard reagents for quantification were purchased from Sigma-Aldrich (St. Louis, MO, USA).

### Statistical Analysis

All experimental data were presented as mean values ± standard deviation (SD). To evaluate data homogeneity, Levene’s test was performed using IBM SPSS Statistics 25 software (IBM Corp., Armonk, NY, USA). One-way analysis of variance was conducted if data variation was homogeneous. When significance was determined, mutual significance within the groups was evaluated using Dunnett’s *t*-test and Duncan’s multiple-range test. Differences in microbial diversity among the groups were assessed using the Wilcoxon rank-sum test. Statistical significance was set at *p* < 0.05. We employed the LEfSe method, which uses the LEfSe to determine the impact of taxa. Microbiotas displaying differential expression between groups were defined as those with an LDA score > 4 and *p*-value < 0.05.

## Results

### Effects of ID-D01 on Body Weight, Food Intake, Water Consumption, and Organ Parameters

There were no statistically significant differences observed for body weight changes between the ID-D01-treated (G3 and G4) and control (G1 and G2) groups (Fig. 1b). The mean food and water consumption values were presented for all groups and showed no statistically significant differences (Tables S1 and S2).

Relative organ weights, including those of the GCM, liver, spleen, kidneys, lungs, and heart, were not significantly different between the control (G1 and G2) and probiotic treatment (G3 and G4) groups (Fig. 1c).

AST and ALT levels were measured to assess hepatotoxicity in each group two days after exhaustive exercise as compared with their steady-state serum levels. Notably, the AST and ALT levels tended to decrease in the exercise groups (G2-G4) compared with that in the non-exercise group (G1) (Fig. 1d). Therefore, our results suggest that oral administration of ID-D01 does not affect body weight, dietary habits, or various organs, such as the GCM, liver, spleen, kidney, lung, and heart.

### Effect of ID-D01 on Maximum Running Distance

**Fig. 2:**
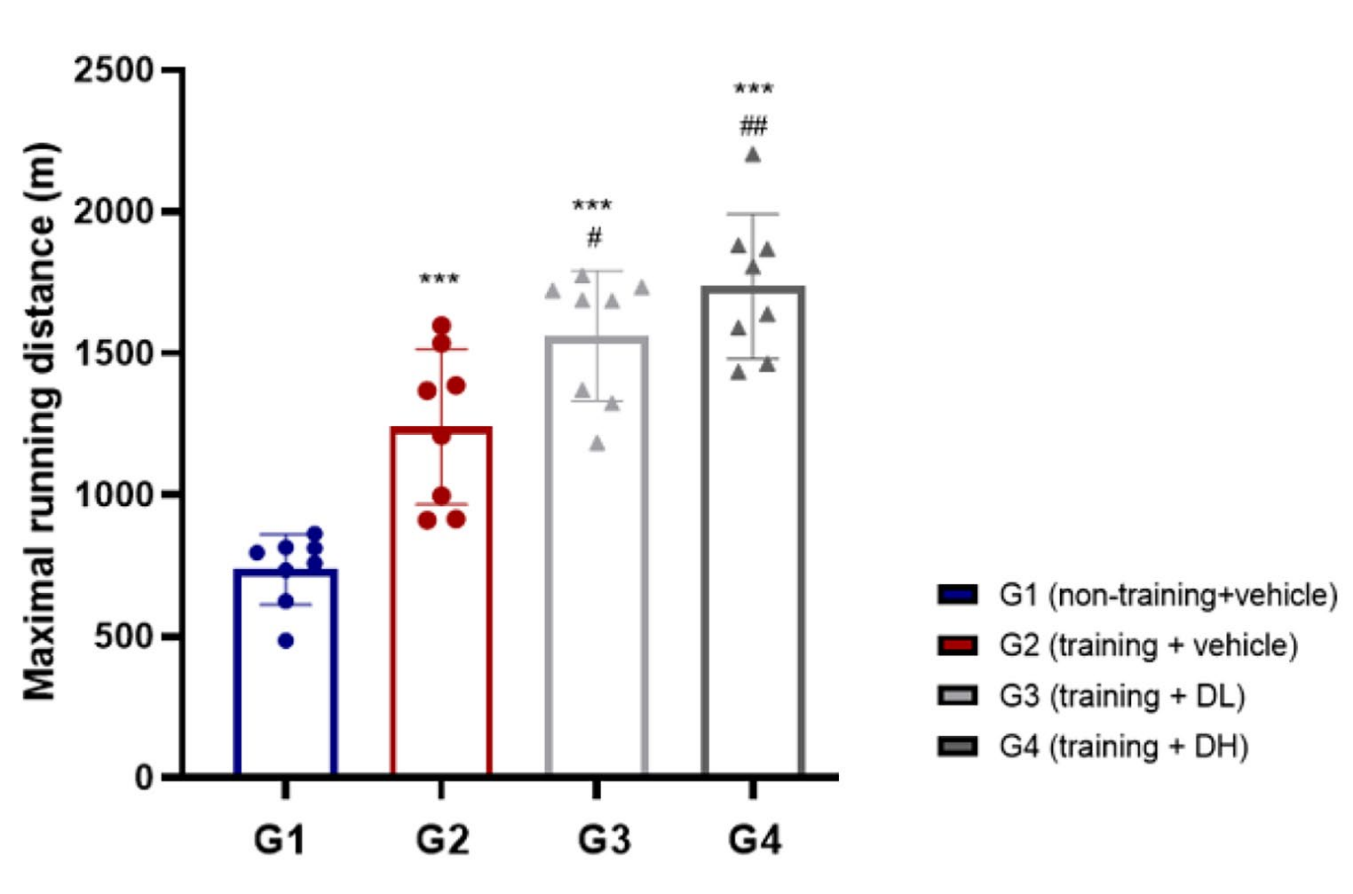
Effect of oral probiotic *Limosilactobacillus reuteri* ID-D01 on the maximal running distance in endurance-exercising rats. Values are presented as mean ± SD (*n* = 8). Significant differences from G1 are denoted as ***p < 0.001 and those from G2 as #*p* < 0.05 and ##*p* < 0.01. DH = high dose; DL = low dose

A graded treadmill exercise model was used to evaluate the effects of ID-D01 probiotic administration on endurance. Treadmill tests are useful for studying physiological adaptations to acute or chronic physical activity and for measuring exercise performance in animals [27]. Fig. 2 shows a significant increase in exercise distance in all training groups (G2-G4) compared with that in the non-training group (G1) (*p* < 0.001). This suggests that graded exercise training was effective in increasing the exercise capacity of G2-G4. In addition, there was a significant improvement in the maximum running distance (m) as a function of dose, with G3 (1560.72 ± 229.32) and G4 (1736.18 ± 255.73) showing notable progress compared with that of G2 (1240.21 ± 274.32). These results indicate that ID-D01 is beneficial for improving endurance.

### Effect of ID-D01 on Fatigue Markers in Serum

**Fig. 3:**
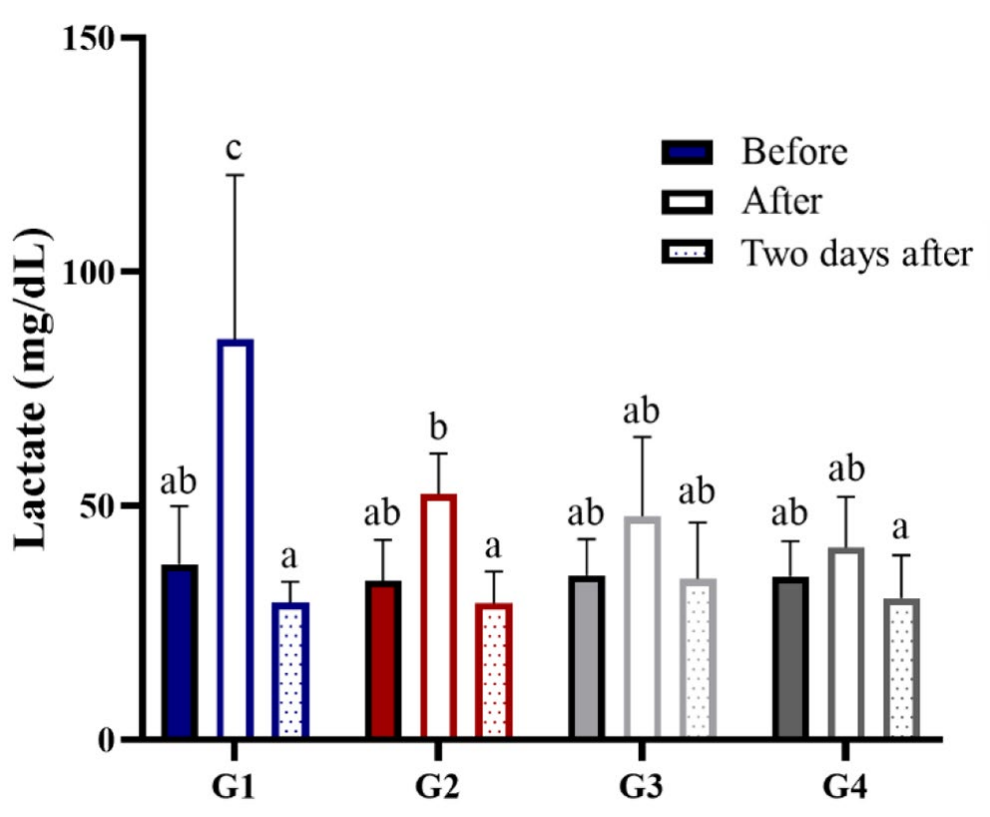
Effect of oral probiotic *Limosilactobacillus reuteri* ID-D01 on the serum lactate profile in endurance-exercising rats. Data are expressed as mean ± SD for rats per group (*n* = 8). Different superscript letters (**a**, **b**, and **c**) indicate significant differences at *p* < 0.01. G1 = non-training + vehicle; G2 = training + vehicle; G3 = training + low dose; G4 = training + high dose

To investigate the changes in blood factors related to fatigue, we analyzed the LDH, CPK, GLU, BUN, CREA, LAC, and ammonia levels in serum samples collected before, after, and two days after the exhaustive exercise program. Before exercise, no significant differences were found between the groups (G1-G4) except for their serum GLU, BUN, and CREA levels, which are shown in Table S3. After exercise, there were no significant differences in the serum LDH, CPK, CREA, and LAC levels within each group when compared with those of G1 or G2 (Table 1). GLU levels decreased significantly in G2 (*p* < 0.01), G3 (*p* < 0.01), and G4 (*p* < 0.001) compared with those in G1, whereas BUN levels increased significantly in G2 (*p* < 0.01), G3 (*p* < 0.001), and G4 (*p* < 0.001) compared with those in G1. Ammonia levels decreased significantly compared with those in G1 (*p* < 0.001). Two days after exercise, serum LDH, GLU, BUN, CREA, LAC, and ammonia levels were not significantly different between each group when compared with those of G1 or G2 (Table 2). However, serum CPK levels were significantly reduced in G3 and G4 compared with those in G1 (*p* < 0.01) and G2 (*p* < 0.01). In addition, pre- and post-exercise LAC production was significantly lower in the probiotic groups than that in the control group (Fig. 3).

**Table 1:** Serum LDH, CPK, GLU, BUN, CREA, LAC, and ammonia levels after exhaustive exercise

Group^a^	LDH (IU/L)	CPK (IU/L)	GLU (mg/dL)	BUN (mg/dL)	CREA (mg/dL)	LAC (mg/dL)	Ammonia (µmol/L)
G1	367.28	501.68	223.93	20.46	0.82	85.55	179.13
	± 109.32	± 325.15	± 31.76	± 2.68	± 0.06	± 35.18	± 64.83
G2	380.50	657.81	176.69^**^	27.39^**^	0.96	52.55	105.00^***^
	± 377.66	± 591.99	± 13.65	± 4.90	± 0.15	± 8.57	± 17.22
G3	301.75	627.83	177.49^**^	27.66^***^	0.98	47.71	106.25^***^
	± 128.00	± 591.86	± 21.39	± 2.26	± 0.11	± 17.00	± 18.34
G4	371.85	345.71	168.38^***^	30.81^***^	0.95	41.09	96.13^***^
	± 175.62	± 329.12	± 20.70	± 4.10	± 0.17	± 10.80	± 22.52

^a^G1: Non-training + vehicle; G2: Training + vehicle; G3: Training + low dose (DL); G4: Training + high dose (DH). Values present the means ± SD (*n* = 8). Significant differences from G1 are denoted as **p < 0.01 and ***p < 0.001. Lactate dehydrogenase (LDH), creatine phosphokinase (CPK), glucose (GLU), blood urea nitrogen (BUN), creatinine (CREA), Lactate (LAC)

**Table 2:** Serum LDH, CPK, GLU, BUN, CREA, LAC, and ammonia levels two days after exhaustive exercise

Group^a^	LDH (IU/L)	CPK (IU/L)	GLU (mg/dL)	BUN (mg/dL)	CREA (mg/dL)	LAC (mg/dL)	Ammonia (µmol/L)
G1	1259.59	733.43	153.23	24.46	0.60	29.29	115.00
	± 954.71	± 453.93	± 13.92	± 17.30	± 0.24	± 4.49	± 22.80
G2	915.95	668.00	150.86	26.29	0.55	29.21	112.75
	± 338.60	± 270.84	± 11.75	± 3.46	± 0.05	± 6.74	± 11.47
G3	865.01	421.10^** ##^	144.19	33.11	0.65	34.39	102.75
	± 782.44	± 231.86	± 20.19	± 35.99	± 0.29	± 12.06	± 13.98
G4	429.60	261.14^** ##^	149.30	19.11	0.52	30.21	96.88
	± 196.62	± 92.17	± 14.31	± 2.35	± 0.05	± 9.22	± 10.48

^a^G1: Non-training + vehicle; G2: Training + vehicle; G3: Training + low dose (DL); G4: Training + high dose (DH). Values are presented as means ± SD (*n* = 8). Significant differences from G1 are denoted as **p < 0.01, and those from G2 as ##*p* < 0.01

### Effect of ID-D01 on Fatigue and Performance Enhancing Factors in Muscle

**Fig. 4:**
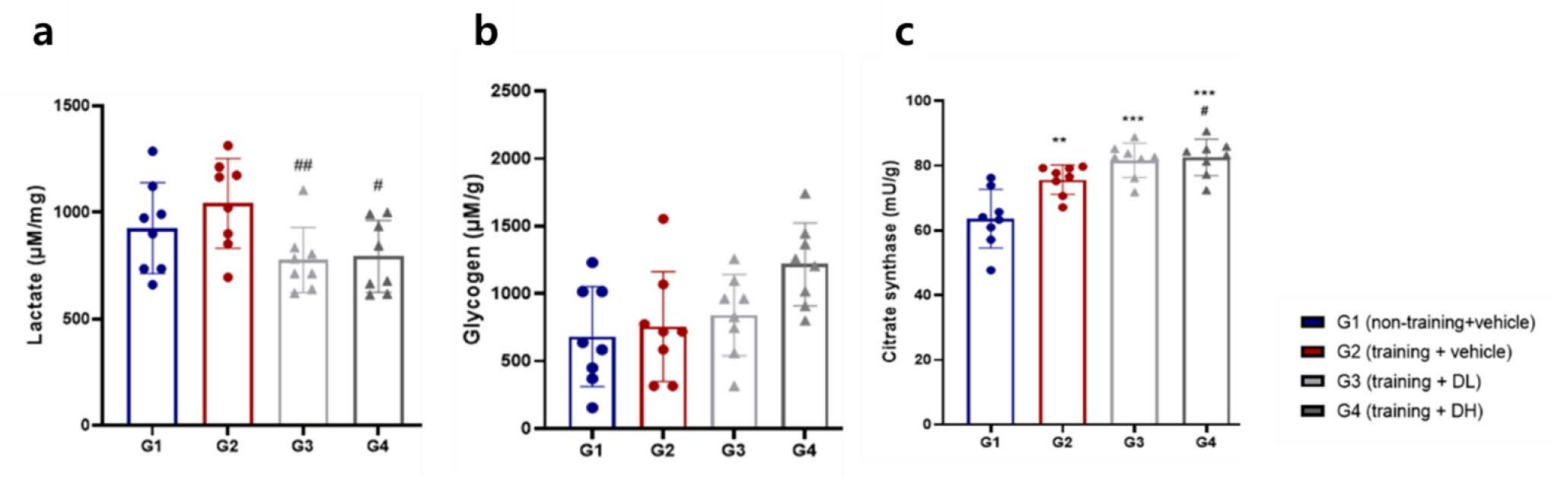
Effects of oral probiotic *Limosilactobacillus reuteri* ID-D01 on lactate (**a**), glycogen (**b**), and citrate synthase (**c**) levels in the gastrocnemius muscles of endurance-exercising rats. Values are presented as mean ± SD (*n* = 8). Significant differences from G1 are denoted as **p < 0.01 and ***p < 0.001, and those from G2 as #*p* < 0.05 and ##*p* < 0.01. DH = high dose; DL = low dose

To evaluate the effect of ID-D01 on endurance, we analyzed indicators of fatigue (LAC) and performance enhancement (glycogen and CS) in GCM tissue. As shown in Fig. 4a, there was a significant decrease in LAC levels in G3 (*p* < 0.01) and G4 (*p* < 0.05) compared with those in G2. Fig. 4b shows that the glycogen content increased in the order of G1 < G2 < G3 < G4, with no significant difference between each group compared with that of G1 or G2. Glycogen, a vital energy reservoir, enhances athletic development and muscle performance by providing energy for muscle contraction. Adequate glycogen levels positively affect endurance and stamina, thereby promoting prolonged exercise.

Fig. 4c shows a significant increase in CS activity in G2 (*p* < 0.01), G3 (*p* < 0.001), and G4 (*p* < 0.001) compared with that in G1. Notably, G4 showed a greater increase in CS activity (*p* < 0.05) than that in G2, indicating that high doses of ID-D01 (G4) increased intramuscular CS activity.

### Effect of ID-D01 on Gut Microbiota and Short-chain Fatty Acid Production

To assess the effect of ID-D01 on gut microbiota, fecal samples were collected from rats eight weeks after treatment and subjected to 16S rRNA gene sequencing analysis.

**Fig. 5:**
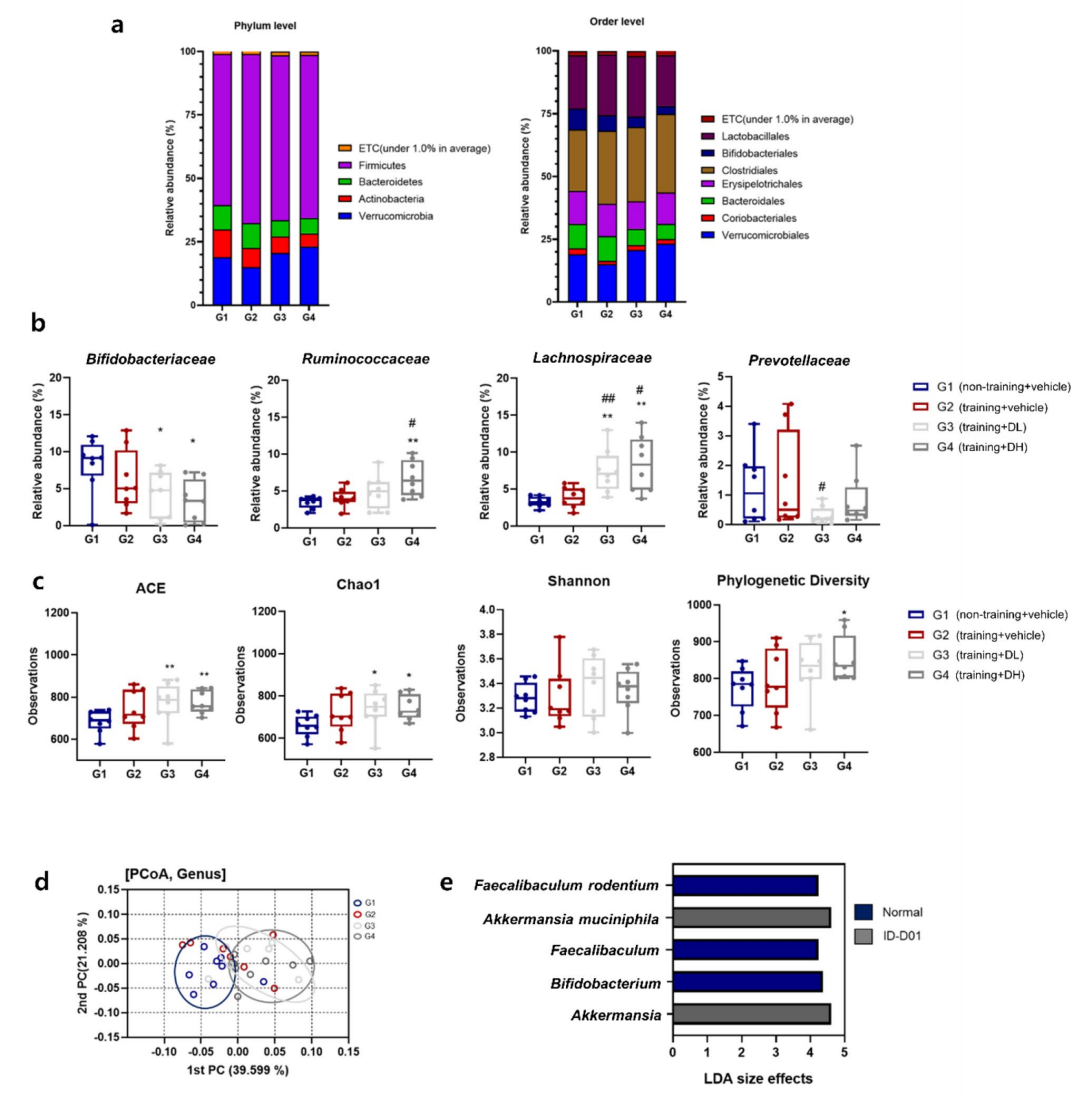
*Limosilactobacillus reuteri* ID-D01 modulates the composition of the gut microbiome. (**a**) Cumulative bar chart of the average relative abundance of bacterial taxa at the phylum and order levels. (**b**) Box plots of relative abundance (%) of the four dominant families in the fecal groups. (**c**) Alpha diversity was analyzed with ACE, Chao1, Shannon, and phylogenetic diversity indices. (**d**) Beta diversity was represented by principal coordinate analysis (PCoA) plots using the Jensen-Shannon dissimilarity metric. (**e**) Taxonomic levels from genus to species (linear discriminant analysis [LDA] score > 4, *p* < 0.05). Horizontal bars represent the effect size for each taxon. Significant differences from G1 are denoted as *p < 0.05 and **p < 0.01, and those from G2 as #*p* < 0.05 and ## *p* < 0.01, DL = low dose; DH = high dose

At the phylum level (Fig. 5a), the abundance of Verrucomicrobia was significantly higher in G4 compared with that in both G1 (*p* < 0.05) and G2 (*p* < 0.05). In contrast, Actinobacteria were less abundant in G4 than in G1 (*p* < 0.05). Additionally, Firmicutes exhibited a significant increase in abundance in G2 compared with that in G1 (*p* < 0.05). At the order level, Clostridiales were significantly more abundant in G4 than in G1 (*p* < 0.05). The abundance of Bifidobacteriales was significantly lower in G3 (*p* < 0.05) and G4 (*p* < 0.05) than that in G1.

At the family level (Fig. 5b), *Bifidobacteriaceae* was more abundant in G1 than in G3 (*p* < 0.05) and G4 (*p* < 0.05), whereas *Ruminococcaceae* had a higher abundance in G4 than in G1 (*p* < 0.01) and G2 (*p* < 0.05). The abundance of *Lachnospiraceae* was significantly increased in G3 and G4, which were treated with ID-D01, when compared with that in G1 (*p* < 0.01) and G2 (*p* < 0.01 and *p* < 0.05, respectively). Furthermore, G3 exhibited a lower abundance of *Prevotellaceae* than that in G2 (*p* < 0.05).

To objectively assess the impact of ID-D01 administration on microbial composition, alpha and beta diversity analyses were conducted. As shown in Fig. 5c, the ACE and Chao1 indices showed significantly higher species richness in the ID-D01-administered groups compared with that in G1 (*p* < 0.01 and *p* < 0.05, respectively). G4 exhibited a noteworthy increase in phylogenetic diversity compared with that of G1 (*p* < 0.05).

To analyze differences in the microbial communities among the four groups (G1-G4), beta diversity was calculated using PCoA and permutational multivariate analysis of variance with the Jensen-Shannon dissimilarity metric. PCoA was used to evaluate the diversity of the gut microbiota at the genus level. As shown in Fig. 5d, significant differences in gut microbiota composition were observed between G1 and G3 (*p* = 0.023), as well as G4 (*p* = 0.012). Additionally, significant differences were observed between G2 and G4 (*p* = 0.006).

LEfSe was used to analyze the core microbial markers representing each microbial community owing to differences in their relative abundance. Notable genus-level microbial variation was observed among ID-D01-treated cohorts, particularly for *Akkermansia* (LDA = 4.60, *p* = 0.01), with an LDA score of 4.0 or more used as the effect size. Significant increases in abundance were observed at the species level, including for *Akkermansia muciniphila* (LDA = 4.60, *p* = 0.01). In contrast, the control group (G1) that did not exercise exhibited the presence of *Bifidobacterium* (LDA = 4.37, *p* = 0.047) and *Faecalibaculum* (LDA = 4.24, *p* = 0.04) at the genus level and *Faecalibaculum rodentium* (LDA = 4.24, *p* = 0.04) at the species level (Fig. 5e).

**Fig. 6:**
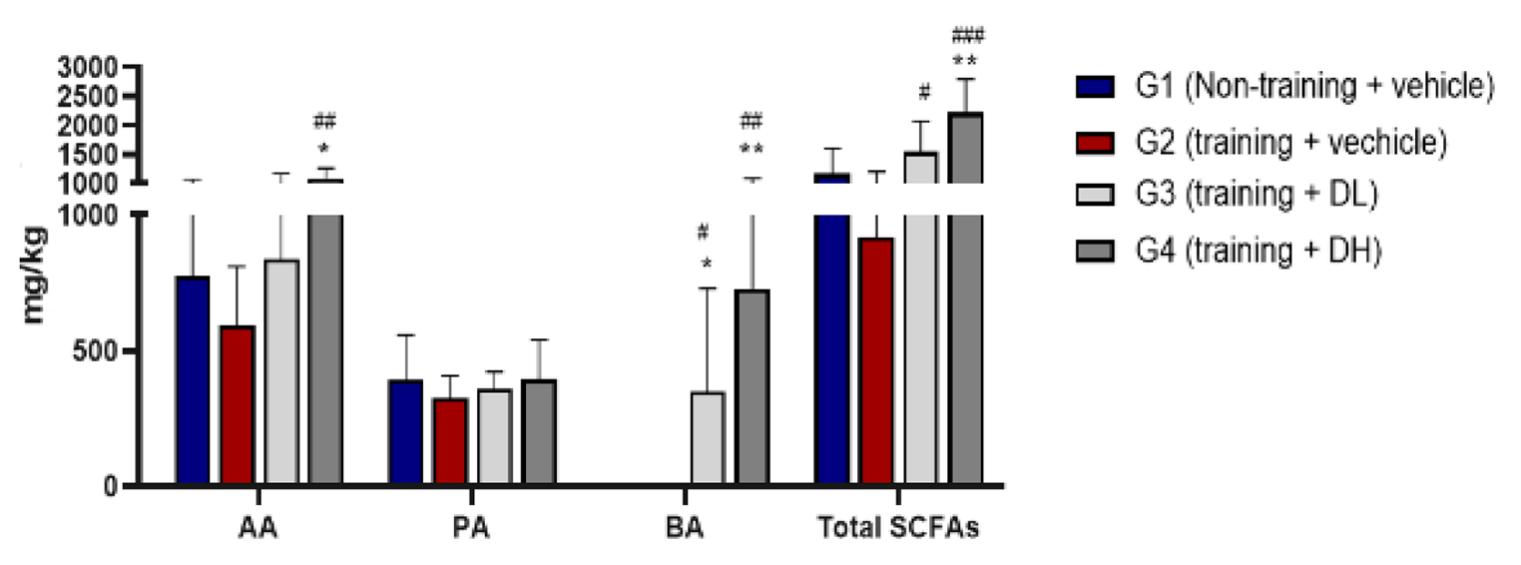
Effects of ID-D01 on short-chain fatty acid (SCFA) production in endurance-exercising rats. Values are presented as mean ± SD (*n* = 8). Significant differences from G1 are denoted as *p < 0.05 and **p < 0.01, and those from G2 as #*p* < 0.05, ##*p* < 0.01, and ###*p* < 0.001. AA, acetate; PA, propionate; BA, butyrate; DL, low dose; DH, high dose

Analysis of SCFAs in feces revealed that oral administration of ID-D01 resulted in elevated production of acetate, butyrate, and total SCFAs in the gut. Notably, acetic acid levels showed a significant increase in G4 compared with those in G2 (*p* < 0.01), and butyrate levels showed a significant increase in G3 (*p* < 0.05) and G4 (*p* < 0.01) when compared with those in G2. Total SCFAs showed a significant increase in G3 (*p* < 0.05) and G4 (*p* < 0.001) compared with those in G2 (Fig. 6).

## Discussion

Blood LAC concentration, a key indicator of anaerobic metabolism during exercise, is used to assess the degree of fatigue [28]. Intramuscular LAC levels are reported to be 2–3 times higher than those in blood, suggesting the potential impact of intramuscular LAC production [28, 29]. In this study, the concentration of blood LAC after exhaustive exercise increased in G1-G2, whereas that in the ID-D01 intake group (G3-G4) was maintained at levels similar to those before exercise. Intramuscular LAC levels in the ID-D01 intake group (G3-G4) were lower than those in G1 and G2, with a significant effect observed after ID-D01 DH intake (G4). The maximal lactate steady state, which indicates the highest exercise intensity that can be sustained without an increase in blood LAC levels, is a key indicator of endurance performance [30–32]. A lower post-exercise LAC concentration is associated with an increased LAC threshold. In this study, the relatively low LAC production observed in the ID-D01 intake groups (G3-G4) suggests that ID-D01 effectively reduced muscle fatigue at the same exercise intensity, contributing to improved endurance performance. CPK levels in the bloodstream are important clinical biomarkers of muscle damage, myopathies, severe muscle breakdown, myocardial infarction, acute renal failure, etc. High-intensity exercise induces physical and chemical damage to muscle tissues [33]. Increased CPK is observed in muscles under hypoxia, where metabolic byproducts accumulate during exercise, resulting in impaired exercise performance [34]. Therefore, elevated CPK levels indicate muscle damage and impaired exercise performance. In this study, immediately after exhaustive exercise, CPK levels in G1, G2, and G3 significantly increased compared with those of pre-exercise levels. Furthermore, we found that CPK levels in only G3 returned to pre-exercise levels two days after exercise. G4 exhibited a different pattern, with CPK levels not increasing immediately after exercise compared with those of pre-exercise levels. Two days after exercise, CPK levels in G4 decreased compared with those in G1 and G2, suggesting less muscle damage and improved exercise performance in G4 compared with those of the other groups (G1-G3).

Increased muscle glycogen storage capacity effectively increases exercise endurance, delays post-exercise fatigue, and accelerates recovery [35]. We observed a trend toward increased intramuscular glycogen content in the probiotic treatment groups (G3-G4), although this was not statistically significant. CS is a major indicator of skeletal muscle oxidative capacity. It catalyzes the formation of citrate from oxaloacetate and acetyl-CoA in the tricarboxylic acid cycle and plays a key role in the regulation of energy-producing metabolic pathways [36, 37]. In addition, increased activity of muscle CS is known to enhance the oxidative capacity in muscle metabolism and delay muscle fatigue [35]. Acetyl-CoA produced from acetate may be preferentially used for citrate synthesis. In addition, acetic acid improves the endurance of exercise-trained mice by increasing skeletal muscle oxidation capacity [38]. Acetic acid-containing SCFAs have been reported as potential regulators of substrate metabolism and skeletal muscle function [39]. In this study, intramuscular CS and acetate levels increased in the ID-D01-administered group (G3-G4), suggesting that the ID-D01 strain contributes to endurance exercise performance by improving muscle oxidative capacity.

Recently, several studies have reported the effects of probiotic intake and exercise performance. The administration of *Lactobacillus plantarum* TWK10 improves endurance in adults [40]. *Veillonella* is abundant in the gut of marathon runners, and it has been demonstrated that *V. atypica* injected into rodents increases endurance [41]. In the present study, we observed an increase in the maximal exercise distance in the ID-D01 oral treatment groups, confirming its effect on improved endurance exercise performance. Furthermore, microbiome analysis of feces after eight weeks of ID-D01 supplementation altered the gut microbiota composition. *Akkermansia* abundance increased in the ID-D01 treatment group, which is consistent with the results of previous studies. *Akkermansia* is often found in the gut of endurance cyclists and its abundance increases during exercise training [42]. Previous studies have shown that administration of human-derived *L. plantarum* Tana increases SCFA production and exercise capacity and decreases fatigue-related biochemical markers [43]. Furthermore, prebiotics (hyaluronan) have anti-fatigue effects, and fatigue indices (such as liver and muscle glycogen) are correlated with SCFA content and antioxidant levels [44]. In our study, ID-D01 increased intestinal acetate, butyrate, and total SCFA production, suggesting that it likely influences exercise performance and fatigue reduction through changes in the gut microbiota and production of SCFAs, which are key metabolites for maintaining colon health [45].

Despite these findings, this study had a few limitations. The first is the low predictive power of the animal models used. Symbiosis between the gut microbiota and host (human) is complex, and interspecies differences in physiology make predicting ID-D01 efficacy in human athletes difficult. Therefore, human trials are necessary to demonstrate the effectiveness of *L. reuteri* ID-D01 on sports performance. Second, because we focused only on the ID-D01 strain, future applied research on optimal dosage and synergistic combinations is needed. In addition, 53 days of administration is not sufficient to investigate the long-term effects of probiotics; further research is needed to understand how *L. reuteri* ID-D01 administration contributes to improved exercise performance. For example, future studies should assess the roles of GPR41 and GPR43, which act as SCFA receptors in skeletal muscle. In addition, activation of AMP-activated protein kinase (AMPK; promotes catabolism to produce ATP) and PGC1-alpha (regulates mitochondrial biogenesis and function) may affect endurance exercise performance. Microbial community dynamics may also play a role; analyses of specific bacterial taxa should provide a closer look at the molecular mechanisms that influence endurance.

## Conclusion

During this study, it was observed that an eight-week regimen of ID-D01 supplementation significantly improved endurance performance in Sprague–Dawley rats and significantly reduced markers of fatigue, such as LAC and CPK. These effects may be related to changes in the gut microbiota induced by ID-D01, which could promote various host metabolic phenotypes. ID-D01 has the potential to enhance exercise performance and reduce fatigue levels. Further research is required to elucidate the molecular mechanisms underlying the anti-fatigue activity of ID-D01.

## Electronic Supplementary Material

Below is the link to the electronic supplementary material.

Supplementary Material 1

## Data Availability

All 16S rRNA Illumina amplicon sequencing data presented in this study are available in the Sequence Read Archive of the National Centre for Biotechnology Information (NCBI) with BioProject ID PRJNA1056773. https://www.ncbi.nlm.nih.gov/sra/PRJNA1056773.

## Declarations

The procedure for isolating ID-D01 from human feces was approved by the Institutional Review Board of Seoul National University, Bundang Hospital (IRB accession number: B-1605-345-003). This animal study was conducted with the approval of the Experimental Animal Ethics Committee at Chungbuk National University (Cheongju, South Korea) (approval number: CBNUA-1718-22-01).
