# Supplementary material for: Effects of Limosilactobacillus reuteri ID-D01 Probiotic Supplementation on Exercise Performance and Gut Microbiota in Sprague-Dawley Rats
Source: Probiotics Antimicrob Proteins. 2024 Apr 18;17(5):3056–66. doi: 10.1007/s12602-024-10257-9 (PMC12532743; doi:10.1007/s12602-024-10257-9)
Supplement: Supplementary file 1 — Supplementary Material 1 [file 12602_2024_10257_MOESM1_ESM.docx]

**Table S1.** Effects of ID-D01 on food intake in endurance exercising rats

| Group^1^ | Food intake (g/7 day/cage) | | | | | | | |
| --- | --- | --- | --- | --- | --- | --- | --- | --- |
|  | 1 week | 2 week | 3 week | 4 week | 5 week | 6 week | 7 week | 8 week |
| G1 | 23.71 | 25.50 | 24.33 | 25.01 | 24.69 | 23.90 | 23.77 | 23.31 |
|  | ± 1.46 | ± 1.72 | ± 4.33 | ± 2.44 | ± 2.31 | ± 2.68 | ± 1.59 | ± 1.46 |
| G2 | 23.65 | 26.56 | 25.12 | 25.86 | 23.79 | 24.04 | 23.76 | 23.41 |
|  | ± 1.67 | ± 2.14 | ± 1.69 | ± 2.76 | ± 1.50 | ± 2.64 | ± 1.78 | ± 2.05 |
| G3 | 24.19 | 25.58 | 24.65 | 25.75 | 25.96 | 25.79 | 23.64 | 23.45 |
|  | ± 0.88 | ± 1.09 | ± 0.93 | ± 1.51 | ± 1.80 | ± 2.75 | ± 0.62 | ± 1.25 |
| G4 | 24.59 | 27.48 | 26.21 | 26.93 | 26.21 | 26.73 | 23.45 | 24.14 |
|  | ± 2.97 | ± 1.83 | ± 2.25 | ± 1.78 | ± 1.79 | ± 1.51 | ± 2.47 | ± 1.44 |

^1^ G1: Non-training+vehicle; G2: Training + vehicle; G3: Training+ DL; G4: Training+ DH. Values are means ± SD (n= 8).

**Table S2.** Effects of ID-D01 on water intake in endurance endurance-exercising rats

| Group^1^ | Water intake (g/7 day/cage) | | | | | | | |
| --- | --- | --- | --- | --- | --- | --- | --- | --- |
|  | 1 week | 2 week | 3 week | 4 week | 5 week | 6 week | 7 week | 8 week |
| G1 | 30.89 | 34.70 | 33.49 | 30.99 | 32.94 | 31.31 | 31.20 | 31.44 |
|  | ± 2.38 | ± 4.08 | ± 6.36 | ± 4.20 | ± 4.90 | ± 3.48 | ± 2.60 | ± 3.78 |
| G2 | 30.74 | 38.05 | 34.34 | 36.23 | 34.67 | 34.51 | 34.71 | 33.28 |
|  | ± 2.65 | ± 3.37 | ± 2.67 | ± 4.50 | ± 3.26 | ± 3.84 | ± 3.60 | ± 4.16 |
| G3 | 30.10 | 38.41 | 35.14 | 34.56 | 36.94 | 35.98 | 33.59 | 33.43 |
|  | ± 3.21 | ± 4.20 | ± 3.95 | ± 2.68 | ± 3.75 | ± 2.70 | ± 2.26 | ± 2.73 |
| G4 | 27.88 | 34.31 | 32.76 | 31.54 | 34.36 | 33.40 | 31.35 | 31.10 |
|  | ± 3.31 | ± 1.87 | ± 3.55 | ± 3.74 | ± 4.94 | ± 4.21 | ± 5.59 | ± 6.18 |

^1^ G1: Non-training+vehicle; G2: Training+vehicle; G3: Training+ DL; G4: Training+DH. Values are means ± SD (n= 8).

**Table S3.** Serum levels of LDH, CPK, GLU, BUN, CREA, LAC, and ammonia before exhausting exercise

| Group^1^ | LDH (IU/L) | CPK (IU/L) | GLU (mg/dL) | BUN (mg/dL) | CREA (mg/dL) | LAC (mg/dL) | Ammonia (umol/L) |
| --- | --- | --- | --- | --- | --- | --- | --- |
| G1 | 294.45 | 312.36 | 142.81 | 12.89 | 0.51 | 37.54 | 83.00 |
|  | ± 186.39 | ± 111.89 | ± 13.22 | ± 1.34 | ± 0.02 | ± 12.35 | ± 21.78 |
| G2 | 399.26 | 295.91 | 140.00 | 18.96 ^***^ | 0.55 ^*^ | 34.06 | 82.75 |
|  | ± 395.70 | ± 124.09 | ± 15.56 | ± 2.29 | ± 0.04 | ± 8.64 | ± 8.92 |
| G3 | 353.19 | 350.23 | 122.20 ^*#^ | 18.69 ^***^ | 0.51 ^#^ | 35.8 | 85.25 |
|  | ± 300.55 | ± 319.74 | ± 10.69 | ± 1.10 | ± 0.03 | ± 7.78 | ± 11.84 |
| G4 | 378.63 | 465.14 | 129.74 | 18.18 ^***^ | 0.51 | 34.95 | 74.25 |
|  | ± 132.08 | ± 301.68 | ± 15.09 | ± 1.87 | ± 0.02 | ± 7.51 | ± 18.20 |

^1^ G1: Non-training+vehicle; G2: Training+vehicle; G3: Training+DL; G4: Training+DH. Values are means ± SD (n= 8). The serum samples were analyzed for aspartate transaminase (AST), alanine transaminase (ALT), lactate dehydrogenase (LDH), creatine phosphokinase (CPK), glucose (GLU), blood urea nitrogen (BUN), creatinine (CREA) using an automatic biochemical analyzer (Beckman Coulter AU480 analyzer (Beckman coulter, Germany). The contents of lactate (LAC) and ammonia were measured using a Hitachi 7180 analyzer (Hitachi, Tokyo, Japan). Significantly different from G1 vs. ***p<0.001; from G2 vs. #p<0.05.
